# TPEN, a Well-Known Zinc Chelator, Sequesters Attomolar-Buffered Cellular Cu(I) Through an Oxygen-Dependent Mechanism

**DOI:** 10.1021/acschemneuro.6c00249

**Published:** 2026-08-06

**Authors:** Arielle Nabatilan, Elena Sergeeva, M. Thomas Morgan, Abigail H. Wright, Larry I. Benowitz, Paul A. Rosenberg, Christoph J. Fahrni

**Affiliations:** † School of Chemistry and Biochemistry and Petit Institute for Bioengineering and Bioscience, 1372 Georgia Institute of Technology, Atlanta, Georgia 30332, United States; ‡ F.M. Kirby Neurobiology Center and Departments of Neurology and Neurosurgery, Boston Children’s Hospital Harvard Medical School, Boston, Massachusetts 02115, United States

**Keywords:** TPEN, PSP-2, zinc, copper, axon regeneration, optic nerve injury, redox trapping

## Abstract

The metal ion chelator TPEN is widely used to study the role of mobile zinc in biology; however, its high affinity for other metal ions raises questions about the specificity when interpreting biological effects of TPEN. Using the Cu­(I)-selective chelator PSP-2, we found that the chelation of copper, and not zinc, likely stimulates axon regeneration after optic nerve injury, thus challenging the previous viewpoint that dysregulation of mobile zinc contributes to regenerative failure upon optic nerve damage. Spectroscopic and electrochemical measurements revealed that TPEN sequesters subattomolar buffered Cu­(I) through a novel redox-trapping mechanism, which was corroborated by fluorescence imaging studies with a Cu­(I)-selective probe in live cells. These findings highlight the ambiguity of TPEN-induced biological effects and identify PSP-2 as a versatile tool for dissecting the role of copper in biological processes.

## Graphical abstract

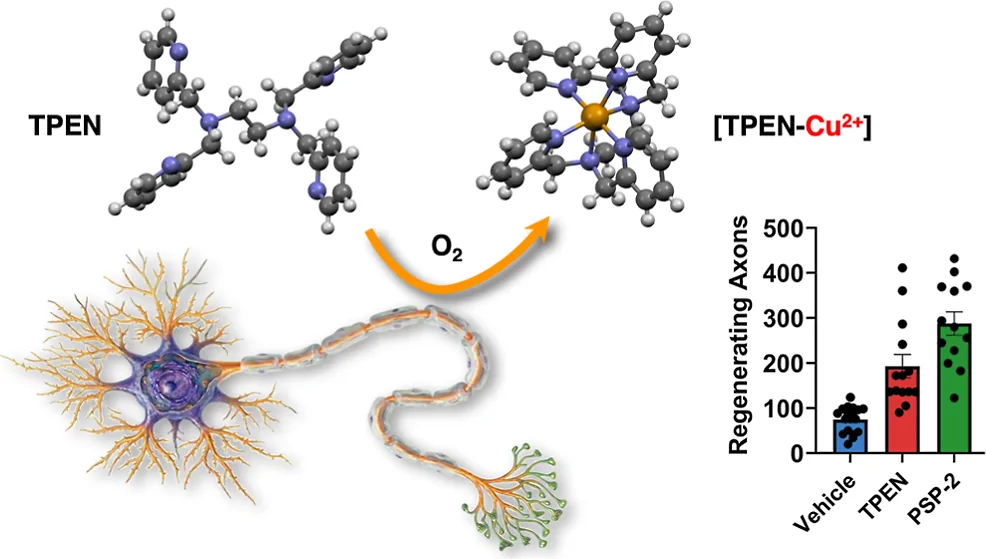

## Introduction

Metal ion-selective chelators are essential tools for manipulating the metal availability in complex biological systems.^1^ Due to its high affinity for Zn­(II), the membrane-permeant chelator *N,N,N′,N′*-tetrakis­(2-pyridylmethyl)­ethylenediamine (TPEN) has found widespread use for elucidating the role of mobile zinc ions in biological processes. Originally reported by Anderegg and Wenk in 1967,^2^ TPEN was first applied to biological studies in 1985 by Tsien and co-workers for probing the interference of heavy metal ions on a Ca­(II)-responsive fluorescent sensor in a tumor cell line.^3^ Yet even though TPEN binds other divalent metal ions with high affinity, it is commonly assumed to affect primarily Zn­(II)-dependent processes. For example, TPEN induces apoptosis in a broad range of cell lines,^4,5^ an observation that has been linked to cellular zinc depletion. Likewise, TPEN impedes germ cell proliferation,^6^ prevents oocytes from entering meiosis,^7^ and inhibits autophagy,^8^ all of which have been tied to TPEN-mediated zinc sequestration.

As a widely employed tool for validating the involvement of zinc, TPEN was also crucial to our studies on elucidating the role of mobile zinc in axon regeneration and retinal ganglion cell (RGC) survival upon optic nerve injury. Administering TPEN via intraocular injection after optic nerve damage increased the survival of RGCs and promoted axon regeneration.^9,10^ We also reported that TPEN suppresses the transient increase of ZnT-3,^9^ a membrane-localized zinc transporter that delivers Zn­(II) into presynaptic vesicles of neurons.^11^ Together, the observed TPEN-induced effects had pointed toward zinc dysregulation as a significant factor in limiting the survival of RGCs and axon regeneration after nerve injury. However, as TPEN does not exclusively bind Zn­(II) with high affinity, we cannot exclude the possibility that the chelator might affect the activity of other d-block metal ions, especially copper. For example, in human keratinocytes, incubation with TPEN depleted both zinc and copper levels by 50% in a dose-dependent fashion, whereas iron levels remained unaffected.^12^ Even at low micromolar concentrations where changes in cellular zinc were insignificant, TPEN reduced copper levels by 15%. Moreover, the TPEN-induced cell death of human colorectal cancer cells was linked to the chelation of intracellular copper rather than zinc.^13^ The increased susceptibility of cancer cells toward TPEN was ascribed to elevated copper levels, which would facilitate intracellular formation of the TPEN–copper complex acting as a potent catalyst for reactive oxygen species generation. Hence, the potential crosstalk with copper-dependent processes underscores the challenges when interpreting TPEN-induced biological effects, despite the presumed preferential interaction of the chelator with mobile zinc compared with copper.

We recently developed a series of high-affinity copper chelators that exhibit an unprecedented selectivity toward copper over other biologically relevant d-block metal ions, including zinc. By combining a phosphine sulfide-stabilized phosphine (PSP) binding motif with a structurally diverse set of ligand architectures, we created a family of chelators that bind Cu­(I) with dissociation constants from the femto- to subzeptomolar range without interference from Zn­(II), Fe­(II), or Mn­(II).^14,15^ Thus, we recognized that the PSP chelators offer a unique opportunity to untangle the potential involvement of copper from zinc-dependent processes in the TPEN-induced axon regeneration in the setting of optic nerve injury.^9^ To this end, we employed here the membrane-permeant chelator PSP-2, which forms with Cu­(I) an air-stable complex with a stability constant of log*K* = 20.0 (*K*_d_ = 10 zM, 0.1 M KCl, 25 °C) without binding Zn­(II), even at millimolar concentrations,^14^ corresponding to a greater than 17 orders-of-magnitude discrimination against Zn­(II) over Cu­(I). Our studies demonstrate that the high-affinity Cu­(I) chelator PSP-2 is as effective as TPEN in improving axon regeneration after optic nerve injury, challenging the previous interpretation regarding the role of zinc dyshomeostasis.^9,16^ This unexpected observation further prompted us to evaluate the ability of TPEN to chelate Cu­(I), the prevalent oxidation state within the reducing intracellular environment, and to explore the redox stability of the resulting complex. Based on these studies, we show that TPEN can sequester Cu­(I) through a redox-trapping mechanism, even when buffered at low zeptomolar concentrations. Consistent with these observations, TPEN also reduced total copper levels in cultured cells and reversed reductively released intracellular Cu­(I) upon incubation with the copper ionophore CuGTSM. These findings may have significant implications for the interpretation of TPEN-induced biological effects attributed to a disruption of zinc homeostasis. Importantly, with the membrane-permeant high-affinity Cu­(I) chelator PSP-2, we identified a versatile tool for untangling the potential role of copper when using TPEN and other putative Zn­(II)-chelators to probe the involvement of zinc in complex biological systems and demonstrate this principle in the case of axon regeneration after injury in the central nervous system (CNS).

## Results and Discussion

### Intraocular Injection of PSP-2 Promotes Axon Regeneration

To investigate whether copper contributes to impeding axon regeneration following CNS injury, we investigated the effect of PSP-2 when administered immediately after optic nerve crush (ONC). For this purpose, we used well validated methods with GAP-43 immunostaining to quantify the number of axons extending 0.5 and 1.0 mm beyond the injury site 2 weeks after ONC (Figure 1A,B). In the injured optic nerve, GAP-43 labels regrowing axons and nerve terminals only and is established as a reliable marker of axonal regeneration.^9,10,17–19^ Intraocular injection of 50 μM PSP-2 resulted in an over 3-fold increase in axon regeneration (Figure 1B, estimated number of GAP-43 positive axons per nerve for PSP-2 at 0.5 mm: 287.6 ± 26.0, at 1 mm: 95.0 ± 10.0) compared to control mice (for vehicle at 0.5 mm: 74.8 ± 6.8; at 1 mm: 38.4 ± 3.6); PSP-2 vs vehicle at 0.5 mm, *p* < 0.0001; PSP-2 vs vehicle at 1 mm, *p* = 0.0001). Remarkably, promotion of regeneration by PSP-2 was comparable to that of TPEN, whose effectiveness has been previously demonstrated^9^ (Figure 1B, estimated number of GAP-43 positive axons per nerve for TPEN at 0.5 mm: 193.2 ± 25.9, at 1 mm: 64.0 ± 6.8; TPEN vs vehicle at 0.5 mm *p* < 0.003; TPEN vs vehicle at 1 mm *p* = 0.0474).

**Figure 1:**
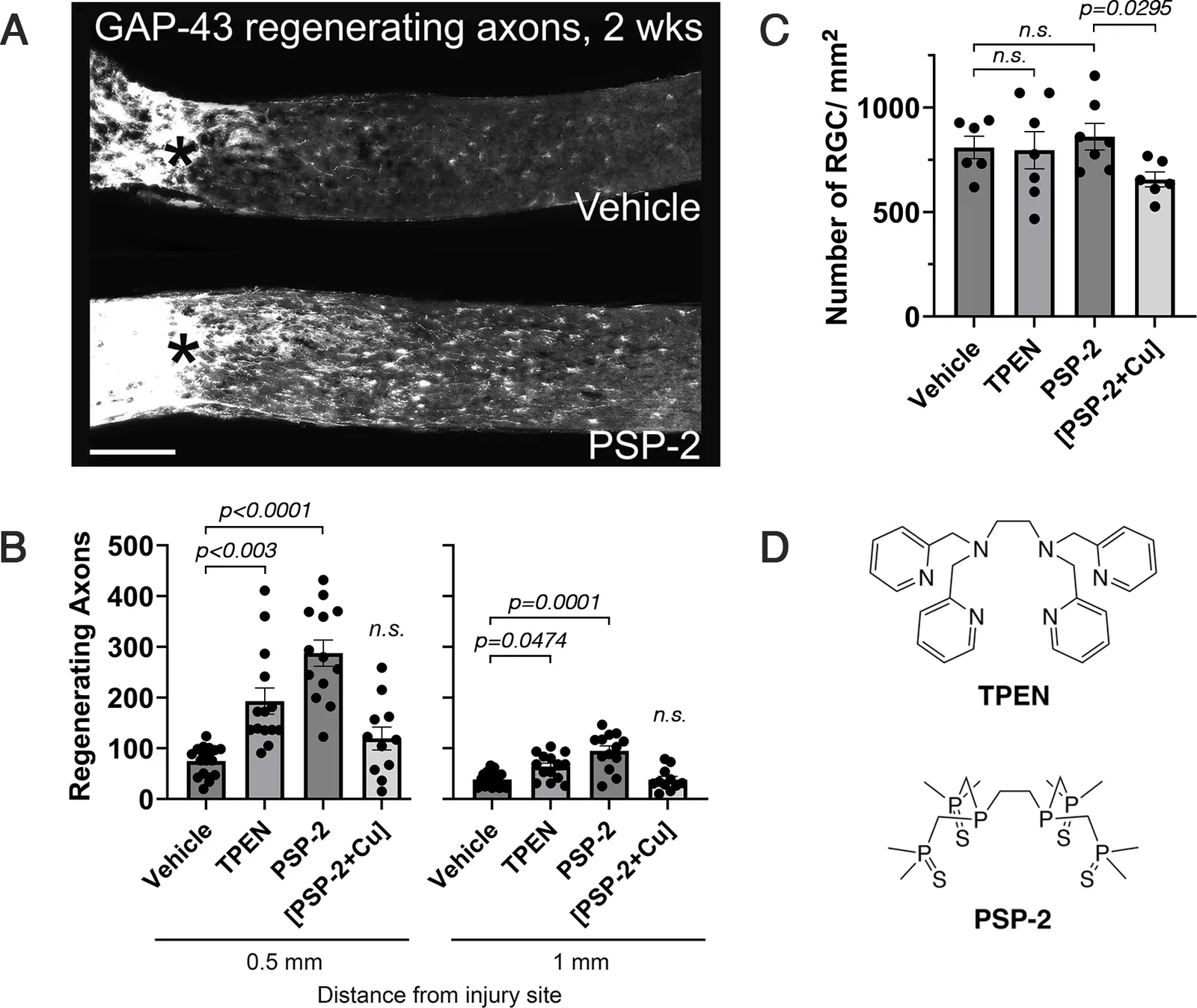
Intraocular injection of the high-affinity Cu­(I) chelator PSP-2 promotes axon regeneration. (A) Longitudinal sections through the optic nerve 2 weeks after ONC and treatment with chelators. Asterisks denote injury site. Scale bar, 200 μm. (B) Quantitation of axon growth at 0.5 and 1 mm distance beyond the crush site 2 weeks after ONC showing the effect of TPEN (100 μM), PSP-2 (50 μM), [PSP-2 + Cu] (50 μM); as a control, PSP-2 was also administered preloaded with [Cu­(I)] compared to Vehicle (1% DMSO in 0.9% saline). Kruskal–Wallis test followed by Dunn’s Multiple Comparisons Test indicated: at 0.5 mm, for TPEN, *p* < 0.003; at 1 mm, *p* < 0.0474; at 0.5 mm for PSP-2, *p* < 0.0001; at 1 mm, *p* = 0.0001; at 0.5 mm, for [PSP-2+Cu], *p* = 0.3436; at 1 mm, *p* > 0.9999. (C) Quantification of RGC survival showing no effect of TPEN (100 μM), PSP-2 (50 μM), compared to Vehicle (Kruskal–Wallis test followed by Dunn’s Multiple Comparisons Test); for [PSP-2+Cu] compared to PSP-2, *p* = 0.0295. All bars show mean ± SEM (D) Line drawings for TPEN and PSP-2.

As PSP-2 does not bind Zn­(II), even at millimolar concentrations, the pro-regenerative effect of PSP-2 can be attributed to Cu­(I)-sequestration. Indeed, when PSP-2 was preloaded with Cu­(I) prior to injection, no significant improvement was observed over control mice (Figure 1B, estimated number of GAP-43 positive axons per nerve for [PSP-2 + Cu] at 0.5 mm: 119.4 ± 22.64, at 1 mm: 38.3 ± 6.6; [PSP-2+Cu] vs vehicle at 0.5 mm, *p* = 0.3436, [PSP-2 + Cu] vs vehicle at 1 mm *p* > 0.9999).

In contrast to their effect on axon regeneration, neither PSP-2 nor TPEN improved retinal ganglion cell (RGC) survival when compared with vehicle (Figure 1C, estimated number of βIII-tubulin positive cells per mm^2^ for PSP-2:861.3 ± 63.6; for TPEN: 796 ± 89 compared to control mice with vehicle: 809.1 ± 53.9; *p* > 0.9999) when administered immediately after ONC). However, when survival in the PSP-2 condition was compared with survival in the PSP-2 + Cu condition (656.2 ± 36), the difference was found to be significant (*p* = 0.0295), suggesting the possibility of a role for copper in the degeneration of RGCs following optic nerve injury.

### Thermodynamic Stability of the TPEN-Cu­(I) Complex

The pro-regenerative effect of PSP-2 implies that TPEN-promoted axon regeneration might also be due to copper chelation. While the stability of the Cu­(II)-TPEN complex is well established,^20^ there are no reports on the TPEN affinity for Cu­(I), the prevalent oxidation state of copper within the reducing intracellular environment. Conventional potentiometric methods employed for the determination of stability constants rely on protonation-induced metal displacement from the ligand;^21^ however, aquated Cu­(I) ions disproportionate at acidic pH or precipitate as sparingly soluble Cu_2_O under neutral or basic conditions. As a workaround, the stability of Cu­(I) complexes can be determined based on a thermodynamic cycle, which relates the stability constant of the corresponding Cu­(II) complex and its reduction potential to the Cu­(I) stability constant and the aqueous Cu­(II/I) redox couple.^22^ The Cu­(I) stability constant can then be determined based on the Nernst relationship (eq 1).

1 $$E^{f}=E_{aq}^{{}^{\circ}}-\frac{2.303RT}{nF}\log\left(\frac{K_{Cu\left(II\right)L}}{K_{Cu\left(I\right)L}}\right)$$

This approach requires that only a single redox-active Cu­(II/I) complex is formed in solution, and that the redox reaction is reversible. Concluding from the published potentiometric data compiled in Table 1,^20^ the 1:1 Cu­(II)-TPEN complex constitutes the sole species over a large pH range (Figure 2A). Although TPEN can form a dinuclear complex with the composition [Cu_2_Cl_2_(TPEN)]^2+^ (logβ_22_ = 26.2, *I* = 0.1, 20 °C) in the presence of 0.1 M chloride ions,^23^ this species is only observed under strongly acidic conditions where a significant fraction of the ligand is protonated, resulting in an excess of Cu­(II) relative to charge-neutral TPEN (Figure 2A). At pH 7.0, TPEN binds Cu­(II) with a remarkably high apparent affinity of log*K* = 20.0 ± 0.1 (*I* = 0.1, 25 °C) to yield the [Cu­(TPEN)]^2+^ complex as the sole species.

**Table 1:** Protonation Constants of TPEN and Thermodynamic Stability Constants of Its Cu­(II)/Cu­(I) Complexes (0.1 M KNO_3_, 20°C)

species	LogK	reference
[HTPEN^+^]/[TPEN][H^+^]	7.19	^20^
[H_2_TPEN^2+^]/[HTPEN][H^+^]	4.86	^20^
[H_3_TPEN^3+^]/[ H_2_TPEN][H^+^]	3.35	^20^
[H_4_TPEN^3+^]/[ H_3_TPEN][H^+^]	2.95	^20^
[Mn(TPEN)^2+^]/[TPEN][Mn^2+^]	10.27 ± 0.06	^20^
[Fe(TPEN)^2+^]/[TPEN][Fe^2+^]	14.61 ± 0.06	^20^
[Co(TPEN)^2+^]/[TPEN][Co^2+^]	16.59 ± 0.15	^20^
[Cu(TPEN)^2+^]/[TPEN][Cu^2+^]	20.56 ± 0.15	^20^
[Cu(TPEN)^+^]/[TPEN][Cu^+^]	16.8 ± 0.1^a^	this work
[Zn(TPEN)^2+^]/[TPEN][Zn^2+^]	15.58 ± 0.12	^20^

a *I* = 0.1 M KCl, 25 °C.

**Figure 2:**
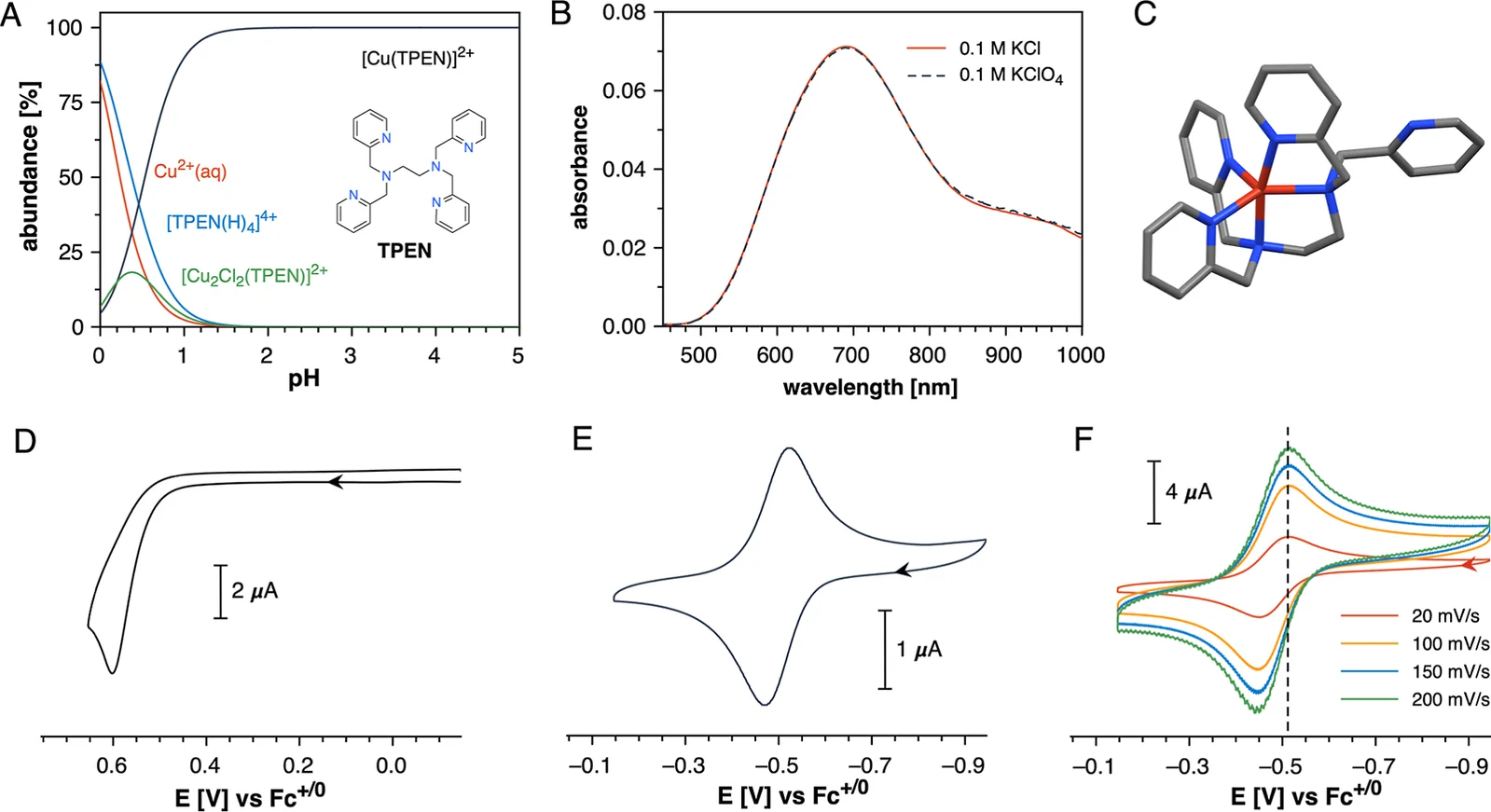
Coordination chemistry, speciation, and electrochemical properties of the Cu­(II)-TPEN complex in aqueous buffer. (A) Speciation diagram for the Cu­(II)-TPEN equilibrium system as a function of pH (derived from the data compiled in Table 1). (B) Absorption spectrum of TPEN (0.6 mM) in the presence of Cu­(II) (0.4 mM) at pH 5.0 (10 mM PIPBS, 0.1 M KCl, 25 °C). (C) Crystal structure of the TPEN-Cu­(II) complex (CCDC code DECDEV, ref 24). (D–F) Cyclic voltammograms of TPEN and its Cu­(II) complex at pH 5.0 (10 mM PIPBS, 0.1 M aqueous KCl, 25 °C; glassy carbon working electrode, Pt counter electrode, aqueous Ag/AgCl/1 M KCl reference electrode, scan rate 100 mV/s, direction indicated by arrows). The potential was referenced against Fc^+/0^ as external standard, measured under the same conditions. (D) Cyclic voltammogram of TPEN (0.6 mM) alone and (E) in the presence of CuSO_4_ (0.4 mM). (F) Scan-rate dependence of the peak-to-peak separation for a mixture of 0.6 mM TPEN and 0.4 mM CuSO_4_.

Consistent with earlier reports,^20^ the absorption spectrum of the [Cu­(TPEN)]^2+^ complex in aqueous buffer revealed a shoulder at longer wavelength relative to the absorption maximum at 691 nm (Figure 2B), indicating formation of a nonsymmetrical penta-coordinated complex in which one of the picolyl arms does not bind to the Cu­(II) center. This observation is in agreement with the reported X-ray structure of [Cu­(II)­(TPEN)]­(PF_6_)_2_,^24^ where the Cu­(II) center assumes a distorted trigonal pyramidal geometry (Figure 2C). Likewise, the X-ray structure of the corresponding Cu­(II) complex with perchlorate counterions assumes a penta-coordinate geometry,^25^ albeit with some notable geometrical differences, suggesting a shallow potential surface that might translate into a fluxional conformational behavior in solution.

To test whether chloride anions might coordinate to the Cu­(II) center in the presence of 0.1 M KCl, we acquired an absorption spectrum in aqueous 0.1 M potassium perchlorate as noncoordinating ionic background. As evident from Figure 2B, the absorption trace in 0.1 M KClO_4_ remains unaltered compared to 0.1 M KCl, indicating that [Cu­(II)-TPEN] does not form a ternary complex with chloride under these conditions. Although 6-coordinate complexes have been described in the solid state,^26^ the dominant species in solution appears to be a 5-coordinate complex, which also accounts for the deviation of the complex stability from the Irving-Williams series as noted by Anderegg and co-workers.^20^

To determine the reduction potential of the Cu­(II/I)-couple bound to TPEN, we next performed a series of cyclic voltammetry studies. Because Cu­(II)-bound inner-sphere water molecules might be subject to hydrolysis at neutral pH, the cyclic voltammetry measurements were performed under mildly acidic conditions.^27^ In addition, we used 0.1 M KCl as the electrolyte to simulate the intracellular ionic background. As evident from the species distribution diagram shown in Figure 2A, the [Cu­(II)-TPEN] complex still remains the sole species under these conditions. Slow-scan cyclic voltammetry of an aqueous buffer solution (pH 5, 10 mM PIPBS, 0.1 M KCl) containing 0.6 mM TPEN alone revealed an irreversible oxidation wave with a peak potential of *E*_p_ = 0.601 V vs Fc^+/0^ (Figure 2D). In the presence of 0.4 mM Cu­(II), a new reversible one-electron redox process was observed with *E*_1/2_ = −0.494 ± 0.004 V vs Fc^+/0^ and a peak separation of 59 mV (Figure 2E), which is consistent with the reversible reduction of TPEN-bound Cu­(II). Increasing the scan rate up to 0.2 V/s had no effect on the peak-to-peak separation, indicating that the redox process is fully reversible (Figure 2F). Given the reversibility of the TPEN-bound Cu­(II/I) couple redox process, the Cu­(I)-stability constant can thus be estimated based on the Cu­(II) stability constant and the reduction potential using Nernst relationship (eq 1). According to IUPAC, the standard potential of the aqueous Cu­(II/I) couple is *E*° = 0.153 V,^28^ which we adjusted to 0.13 V to account for the ion activity at 0.1 M ionic strength.^22,29^ After referencing *E*_1/2_ to SHE (standard hydrogen electrode), we obtained *E*_1/2_ = −0.094 ± 0.004 V. With an apparent affinity of log*K* = 17.90 for Cu­(II) at pH 5.0, eq 1 yields a Cu­(I) stability of log*K* = 14.1, which translates to a pH-independent complex stability constant of log*K* = 16.8 ± 0.1. At physiological pH = 7.4, the apparent Cu­(I) affinity is slightly lower with a log*K*′ of 16.4 ± 0.1.

### Competitive Chelation of Cu­(I) from BCS and PSP-2 by TPEN

To test whether Cu­(I)-sequestration by TPEN might be hampered by a large kinetic barrier, we evaluated the competitive exchange of Cu­(I) with bathocuproine disulfonate (BCS). This bidentate heterocyclic ligand forms with Cu­(I) an orange-colored complex with an absorption maximum at 483 nm (ε = 13,300 M^–1^ cm^–1^). With a logβ_2_ value of 20.80 ± 0.03,^29^ the ligand is well-suited to perform competition titrations of high-affinity Cu­(I) ligands and metalloproteins with *K*_d_ values in the low attomolar to zeptomolar range.^31,32^ Despite the weaker Cu­(I) affinity, TPEN readily removes Cu­(I) from BCS. As illustrated with the UV–vis trace shown in Figure 3A, addition of 50 μM TPEN to a 30 μM solution of the preformed [(BCS)_2_Cu­(I)] complex resulted in greater than 97% removal of Cu­(I) within 3 min. We reasoned that the unexpected metal exchange reaction might involve a change in oxidation state to Cu­(II), which would bind to TPEN with much higher affinity (log*K* Cu­(II) = 20.54)^20^ than BCS (logβ_2_ Cu­(II) = 12.42 ± 0.07).^29^ Physiologically, ambient dioxygen could serve as the oxidant. To test this hypothesis, we repeated the ligand competition experiment in deoxygenated buffer that was purged with argon for 10 min. Under these conditions, we observed an initial drop of the absorbance from 0.34 to 0.25 within 12 s, corresponding to 34% of Cu­(I) transfer from [(BCS)_2_Cu­(I)] to TPEN (Figure 3B, blue trace). In contrast to aerated buffer (Figure 3B, red trace), the metal exchange did not proceed to completion. A simulation of the equilibrium system with the Hyperquad Simulation and Speciation software HySS^32^ revealed that the initial drop is consistent with the measured TPEN Cu­(I) complex stability constant of log*K* = 16.8, which should result in 33% removal of Cu­(I) from [(BCS)_2_Cu­(I)].^29^

**Figure 3:**
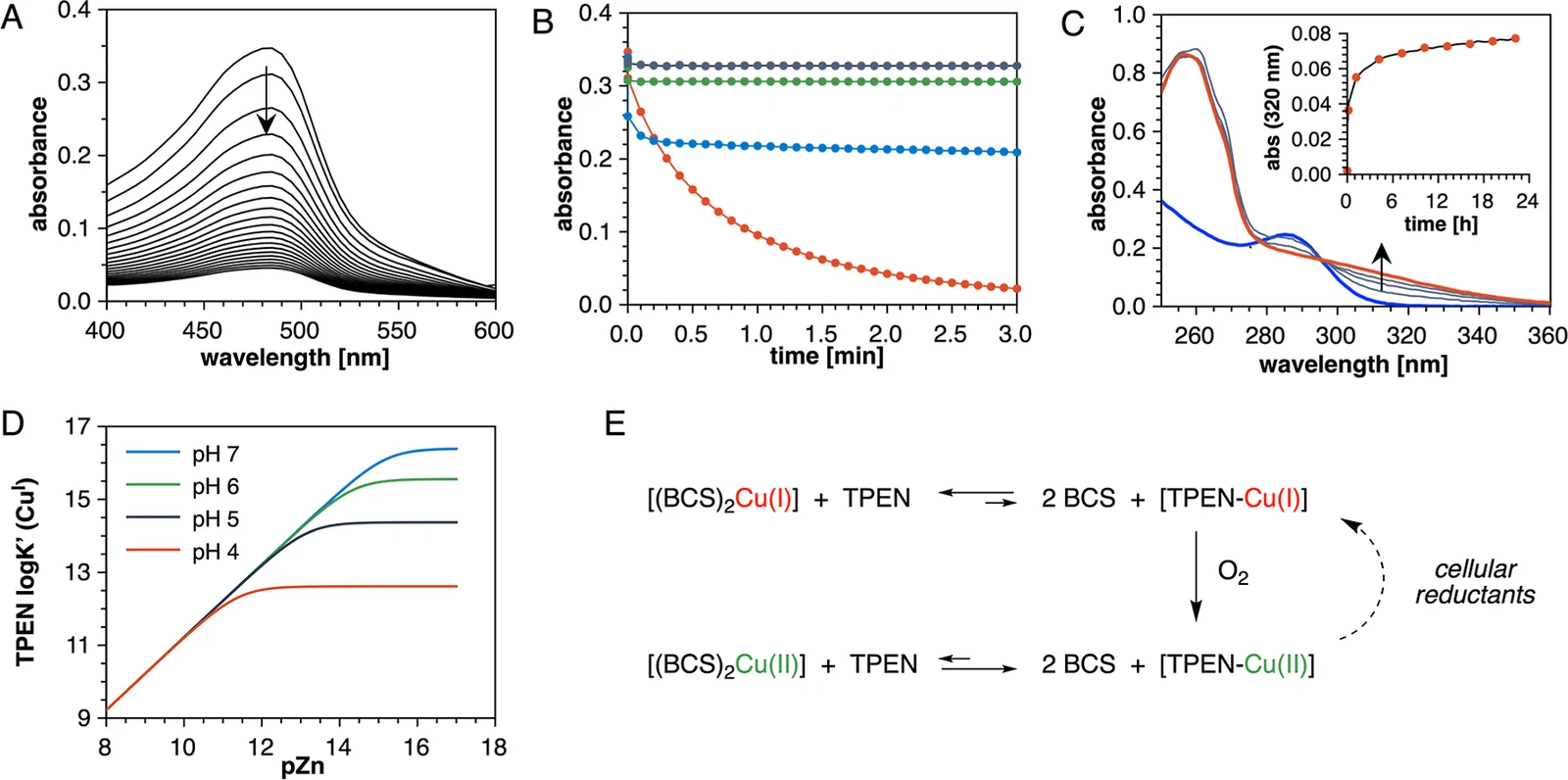
TPEN sequesters Cu­(I) from high-affinity Cu­(I) ligands through an oxygen-dependent mechanism. (A) Time-dependent changes of the UV–vis absorption spectrum of the [Cu­(I)­(BCS)_2_] complex, formed in situ with 100 μM of BCS and 30 μM of [Cu­(I)­MCL-2]­PF_6_ in pH 7.0 buffer (10 mM PIPES, 0.1 M KCl, 25 °C), upon addition of 50 μM TPEN (B) Time-dependence of the absorbance at 483 nm for the [Cu­(I)­(BCS)_2_] complex upon addition of 50 μM TPEN in aerated buffer (red trace), in deoxygenated buffer (blue trace), in Zn­(II)-buffered solution (1 mM EGTA buffer with pZn = 9, green trace), and with TPEN preloaded with an equimolar amount of Zn­(II) (gray trace). (C) Time-dependent changes of the UV–vis absorption spectrum of [(PSP-2)­Cu­(I)]Cl (50 μM) in aerated pH 7.0 buffer (10 mM PIPES, 0.1 M KCl, 25 °C) after addition of 50 μM of TPEN. The blue trace was acquired before TPEN addition and the red trace after equilibration for 24 h. Inset: Time-dependent absorbance change at 320 nm. (D) Apparent Cu­(I) affinity of TPEN as a function of buffered Zn­(II) concentration (pZn) and pH calculated based on eq 2. (E) Redox trapping mechanism of Cu­(II) by TPEN from high-affinity Cu­(I) ligands. Although BCS binds Cu­(I) more tightly than TPEN, the [TPEN-Cu­(I)] complex is readily oxidized under ambient conditions to produce [TPEN-Cu­(II)], which is several orders of magnitude more stable compared to [(BCS)_2_Cu­(II)]. Over time, TPEN is therefore able to sequester BCS-bound Cu­(I) quantitatively.

Within a cellular environment, Zn­(II) might compete for Cu­(I) binding to TPEN and thus lower its ability to sequester Cu­(I) from metalloproteins, even in the presence of dioxygen or other oxidants. To simulate the buffered Zn­(II) availability in the cytosol of mammalian cells,^33^ we utilized 1 mM EGTA as metal buffer ligand and adjusted the total Zn­(II) amount such that the resulting free Zn­(II) activity was kept constant at pZn = 9. With an apparent log*K*′ of 13.2 at pH 7.0, the Cu­(II) affinity of EGTA is almost 7 orders of magnitude lower compared to TPEN (logK′ 20.1 at pH 7.0). Therefore, EGTA is not expected to interfere with the redox trapping mechanism by competitively chelating Cu­(II) under these conditions. Addition of 50 μM TPEN to a pre-equilibrated solution of 30 μM [(BCS)_2_Cu­(I)] complex in aerated EGTA-Zn­(II) buffer almost completely abrogated the metal exchange reaction (Figure 3B, green trace). Based on the average absorbance of 0.31 after equilibration for 3 min, we concluded that only 10% of Cu­(I) was removed from [(BCS)_2_Cu­(I)] even though both Cu­(I) and Cu­(II) bind to TPEN with higher affinity compared to Zn­(II) (Table 1). Thus, the incomplete exchange indicates that the replacement of prebound Zn­(II) by Cu­(I) proceeds with slow kinetics. The initially observed rapid drop might be due to a more favorable rate for Cu­(I) transfer from [(BCS)_2_Cu­(I)] complex compared to Zn­(II) exchange with the EGTA-Zn­(II) buffer. To further explore this possibility, we repeated the competition experiment by adding the preformed TPEN-Zn­(II) complex in lieu of TPEN alone. As expected, the exchange reaction was now completely inhibited under these conditions (Figure 3B, gray trace).

The observed lowering of the Cu­(I) affinity in the presence of Zn­(II) can be expressed by the corresponding apparent stability constant *K*′. Originally introduced by Schwarzenbach to simplify the pH-dependence of stability constants,^34^ the concept can be expanded to account for competitive binding of Zn­(II). Thus, assuming a constant activity of free Zn­(II) at 1 nM or pZn = 9 (with pZn = −log­[Zn­(II)]_free_), the apparent Cu­(I) affinity of TPEN at neutral pH is lowered from log*K*′ = 16.4 to 10.2 (Figure 3D). Even at 1 pM Zn­(II) activity (pZn = 12), the Cu­(I) affinity decreases more than 1000-fold to log*K*′ = 13.2. Upon acidification, Zn­(II) binding remains competitive at least down to pH 4.

To test whether TPEN might be able to sequester Cu­(I) from ligands with even higher affinity than BCS, we performed an additional competition experiment with PSP-2 (log*K* = 20.0, 25 °C). As shown in Figure 3C, addition of 50 μM of TPEN to 30 μM of the [Cu­(I)­(PSP-2)] complex (blue trace) resulted in almost quantitative metal exchange, albeit with much slower kinetics compared to [(BCS)_2_Cu­(I)]. The resulting absorption spectrum is consistent with a reference spectrum of the [Cu­(II)-TPEN] complex, indicating that the metal exchange reaction again proceeded by means of a redox process.

Taken together, TPEN has a remarkable ability to sequester copper from high-affinity Cu­(I) ligands in the presence of oxygen. The spectrophotometric competition studies are consistent with a redox-trapping mechanism, where the initially formed [Cu­(I)­TPEN] complex is rapidly oxidized by dioxygen to yield [Cu­(II)­TPEN] (Figure 3E). Although the formation of [Cu­(I)­TPEN] is incomplete due to the lower affinity of TPEN compared to BCS, subsequent conversion to [Cu­(II)­TPEN] effectively traps Cu­(II) bound to TPEN, which has a much higher Cu­(II) affinity (log*K* = 20.56) than BCS (logβ_2_ = 12.42 ± 0.07, *I* = 0.1, 25 °C).^29^ The slower exchange kinetics observed for PSP-2 is likely due to its higher Cu­(I) affinity over BCS, thus significantly reducing the amount of [Cu­(I)­TPEN] formed in the initial exchange equilibrium. Similar to BCS, the Cu­(II) affinity of PSP-2 is several orders of magnitude lower compared to its Cu­(I) affinity, thus rendering the exchange equilibrium nearly irreversible upon oxidation of [Cu­(I)­TPEN] to [Cu­(II)­TPEN].

### Competitive Copper Chelation by TPEN in 3T3 Mouse Fibroblasts

At neutral pH, a significant fraction of TPEN remains charge-neutral and can readily enter cells to affect a range of metal-dependent biological processes. Increasing evidence suggests that cells maintain a kinetically labile Cu­(I) pool that is tightly buffered in the low attomolar range.^35,36^ Although TPEN has been primarily used to intercept Zn­(II)-dependent processes, the above-mentioned metal competition experiments suggest that the ligand may also be capable of interacting with this tightly buffered Cu­(I) pool. To explore this possibility, we employed our recently developed Cu­(I)-responsive fluorescent probe crisp-17 to visualize TPEN-induced dynamic changes of cellular Cu­(I) in 3T3 mouse fibroblasts. Optimized for two-photon excitation microscopy, crisp-17 changes the fluorescence emission from green to orange upon saturation with Cu­(I) and is thus suitable for ratiometric imaging analysis with two bandpass filters BP1 (479–536 nm, green emission) and BP2 (611–750 nm, orange emission).^35^ Because the total level of cellular copper would be insufficient to sense TPEN-induced changes in Cu­(I) availability under basal conditions, we increased the cellular Cu­(I) pool by preincubation with CuGTSM, a membrane-permeant Cu­(II) thiosemicarbazone complex that releases Cu­(I) upon entering the reducing cellular milieu.^37^ ^a,b^ As shown in Figure 4A, incubation with 10 μM CuGTSM induced a robust increase in the emission ratio (BP2/BP1) from 0.75 to 1.7, reaching a plateau after approximately 10 min. The subsequent addition of 50 μM TPEN elicited a rapid drop of the emission ratio from 1.7 to 0.9 (*p* < 0.0001). Interestingly, this initial decline was followed by a gradual upward drift in the ratio, presumably reflecting the continuous reductive release of Cu­(I) from residual CuGTSM stores. To confirm the overall reversibility of the probe response, we added 50 μM PSP-2, which further reduced the emission ratio to 0.6 (*p* < 0.0001), slightly below the initial value as previously reported.^35^ In a control experiments, we explored the kinetics of Cu­(I) clearance by PSP-2 alone omitting the initial TPEN treatment (Figure 4B). While PSP-2 reduced the crisp-17 fluorescence ratio below the basal baseline to 0.63 (*p* < 0.0001), the initial rate of the ratio decrease was virtually identical to that observed with TPEN, yielding initial slopes of −0.44 ± 0.01 min^–1^ for PSP-2 and −0.45 ± 0.05 min^–1^ for TPEN. Taken together, these experiments demonstrate that TPEN functions as a potent Cu­(I) chelator within the complex intracellular environment. Notably, the ligand is capable of effectively sequestering Cu­(I) from crisp-17 despite exhibiting an apparent affinity that is 5-fold lower.

**Figure 4:**
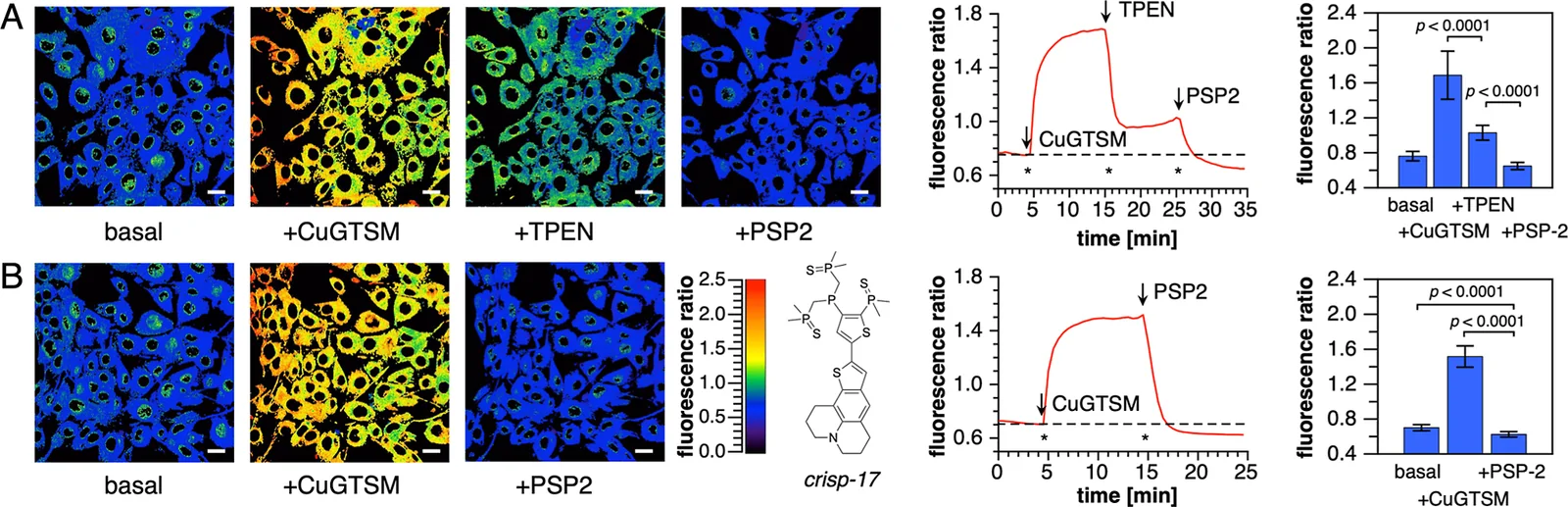
Competitive removal of Cu­(I) in live mouse fibroblasts visualized by two-photon excitation microscopy using the ratiometric fluorescent probe crisp-17. (A, left) Ratio-images before (basal) and after consecutive addition of CuGTSM (10 μM), TPEN (50 μM), and PSP-2 (50 μM). (A, middle) Average intensity ratio as a function of time (*n* = 10). The asterisks mark the time points of the ratio images shown to the left. (A, right) Mean fluorescence ratio of the cytoplasmic region averaged over 10 cells (*p* values calculated for *n* = 10 using a two-tailed test). Raw intensity images were acquired between 479 and 536 nm (BP1) and 611–750 nm (BP2) with two-photon excitation at 880 nm. The false-color micrographs represent the derived ratio-images (BP2/BP1). Scale bar 20 μm. (B) Control experiment carried out as described for A but without TPEN addition.

### TPEN-Induced Changes of Total Transition Metal Levels in 3T3 Mouse Fibroblasts

The ratiometric imaging studies with crisp-17 revealed that TPEN removes Cu­(I) with rapid kinetics, implying an associative mechanism driven by direct intermolecular interactions. To evaluate to what extent transition metals are not only chelated but also depleted from cells, we quantified changes in total cellular levels of Mn, Fe, Cu, and Zn by ICP-MS. To this end, 3T3 mouse fibroblasts were grown to 80% confluency in medium supplemented with 50 μM Cu­(II) and then incubated with 10 μM TPEN for 5 h. After removing the medium, cells were washed and acid-digested for ICP-MS elemental quantification. As expected, TPEN treatment reduced the cellular Zn content by 50% (*p* < 0.0001); however, other trace metal levels were also affected, with a reduction in Mn by 66% (*p* = 0.0002), Fe by 13% (*p* = 0.031), and Cu by 32% (*p* = 0.0006) (Figure 5A). Consistent with earlier studies, the highly selective Cu chelator PSP-2 resulted in a 48% reduction of Cu (*p* < 0.0001), whereas Zn, Fe, and Mn levels remained statistically unaltered.

**Figure 5:**
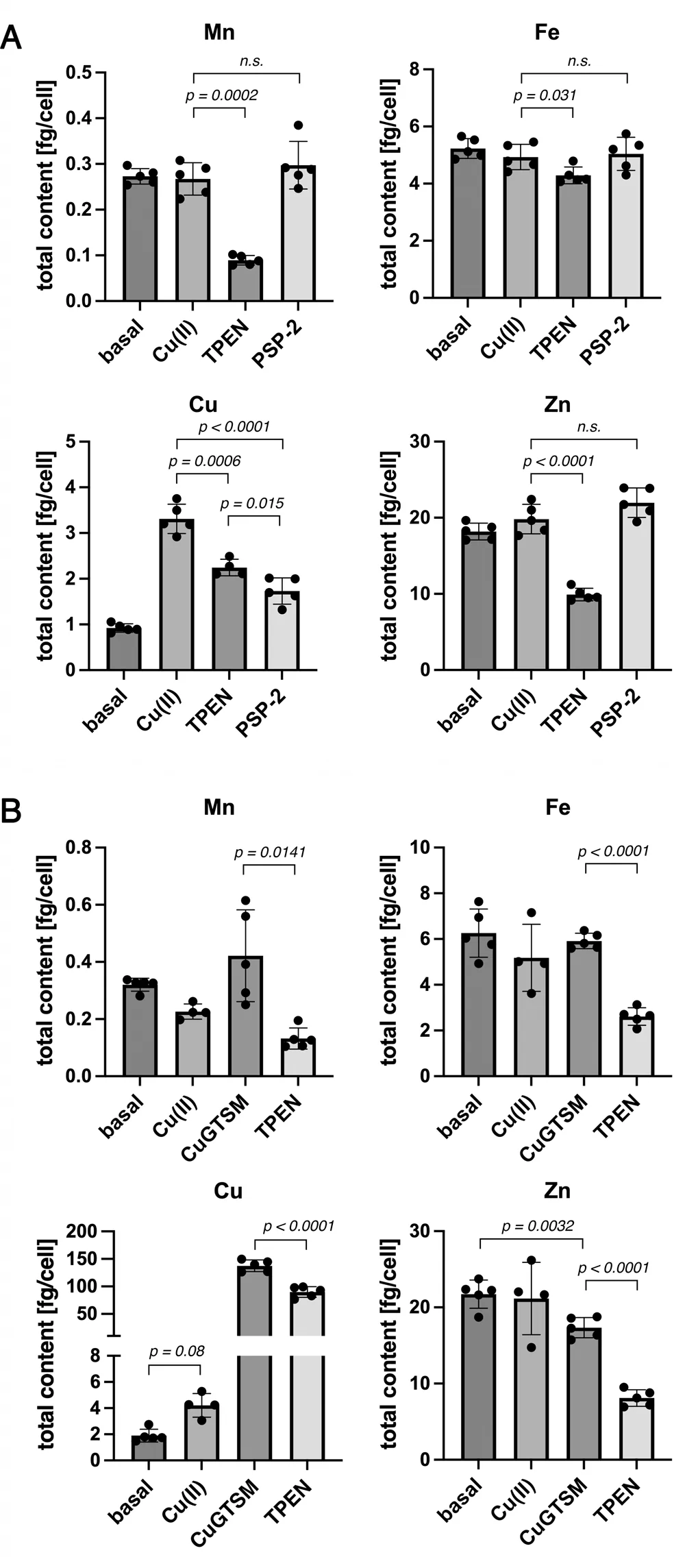
Competitive removal of cellular trace metals by TPEN. (A) ICP-MS quantification of the total cellular Mn, Fe, Cu, and Zn content of 3T3 mouse fibroblasts (average of ∼5 × 10^6^ cells) grown in medium supplemented with 50 μM CuSO_4_·5H_2_O before and after incubation with either 10 μM TPEN or 10 μM PSP-2 for 5 h. The metal content of cells without supplementation (basal) is provided as reference. (B) ICP-MS quantification of the total cellular Mn, Fe, Cu, and Zn contents of 3T3 mouse fibroblasts incubated with 10 μM CuGTSM for 15 min followed by addition of 10 μM TPEN for 5 h. The metal content of cells without supplementation (basal), and supplementation with 50 μM CuSO_4_·5H_2_O (Cu­(II)) is provided for comparison. All *p*-values were calculated for *n* = 5 using a two-tailed test.

To evaluate changes in cellular copper under conditions recapitulating the crisp-17 imaging experiments, cells were preincubated with 10 μM CuGTSM for 15 min prior to exposure to 10 μM TPEN for 5 h (Figure 5B). Preincubation with CuGTSM elevated the cellular Cu content over 70-fold (*p* < 0.0001) relative to basal controls, and over 30-fold compared to cells maintained in Cu­(II)-supplemented medium. Notably, this copper overload induced a 20% decrease in endogenous Zn levels (*p* = 0.0032), suggesting competitive displacement from intracellular binding sites. Consistent with the ratiometric imaging data, subsequent addition of TPEN elicited a marked 35% reduction of total cellular Cu (*p* < 0.0001). Despite the large excess of intracellular Cu, TPEN still sequestered 53% of total cellular Zn (*p* < 0.0001). Collectively, the ICP-MS data establish that TPEN functions as a multitarget chelator that effectively reduces cellular copper levels in addition to its well-known zinc-depleting activity. Importantly, these findings demonstrate that TPEN not only binds to intracellular copper but actively promotes cellular export, thus resulting in substantial depletion even under conditions of severe copper overload.

## Conclusions

The high-affinity Cu­(I)-selective chelator PSP-2 demonstrated axon regeneration-promoting efficacy comparable to, or exceeding, that of TPEN following optic nerve injury. This finding challenges our earlier conclusion that dysregulation of mobile Zn­(II) is a significant cause of regenerative failure, pointing to copper as the relevant cation.^9^ While we cannot exclude the possibility that both TPEN and PSP-2 target a metal-independent pathway, for example by acting as small molecule inhibitors of a critical enzyme, the data strongly suggest a critical role for copper in the regenerative failure upon optic nerve injury. Although the Cu­(I) affinity of TPEN is too low to effectively compete with the attomolar buffering of cellular Cu­(I), the ligand can nevertheless sequester copper through an oxygen-dependent redox-trapping mechanism as demonstrated by spectroscopic studies. Within the reducing cellular environment, the [Cu­(II)­TPEN] complex may engage in redox cycling, which likely constitutes a significant source of TPEN-induced toxicity.^13^ However, at nanomolar Zn­(II) activity, the ability of TPEN to sequester cellular copper would be expected to be attenuated by competitive formation of the Zn­(II)-TPEN complex. Thus, the TPEN-induced toxicity, if indeed mediated by copper, might be suppressed by increasing cellular Zn­(II)-availability. Indeed, studies with Jurkat cells revealed that TPEN-induced apoptosis is independent of its activity as a Zn­(II) chelator, and that the cytotoxicity of TPEN could be reversed through supplementation with Zn­(II).^38^ Given the slow dissociation of Zn­(II) from the Zn­(II)-TPEN complex, the exchange kinetics with metal ions that bind with higher affinity inevitably occurs with slow kinetics. Therefore, traditional metal complementation studies cannot unequivocally establish the identity of the biologically active metal ion,^5,39,40^ as a more abundant inactive metal ion might block sequestration of the biologically active target by the chelator, even in cases where the metal exchange reaction is thermodynamically favorable.

Given the reducing intracellular environment, most copper exists in the reduced Cu­(I) state coordinated by thiol-containing ligands. We assume that the major storage sites for copper, as for zinc, are metallothionein proteins, and that both metals can be released by reactive oxygen or nitrogen species (ROS or RNS) interacting with thiol groups.^41^ In addition, there is evidence that NMDA receptor activation may cause the release of copper from neurons related to the translocation of the copper-transporting ATPase (Atp7a) to the plasma membrane.^41^ The cellular location of the relevant mobile copper pool is unknown, and possibilities include release within or onto RGCs to impede their regeneration capacity or within or onto cells or circuits that interact with RGCs and influence how they respond to axonal injury.

An important question is how the release of copper from intracellular stores might impede axon regeneration. One possibility is that copper release potentiates oxidative stress injury occurring in damaged tissue. Optic nerve crush damages RGC axons, leading to mitochondrial dysfunction and excessive production of ROS, as well as RNS by activation of a recently discovered multicellular signaling pathway.^42,43^ In turn, ROS and RNS promote the indiscriminate release of Cu­(I) from thiol-binding sites, as previously demonstrated using the thiol-selective oxidant 2,2′-dithiodipyridine,^36^ thereby further exacerbating oxidative stress through Fenton-type chemistry. In addition, numerous pathways and processes that might be influenced by increased mobile copper have been shown to play a role in axon regeneration. Inflammatory cells, especially macrophages and neutrophils, regulate the ability of RGCs to regenerate axons through secreted factors.^45^ Copper homeostasis is an important regulator of the function of both of these cell types.^46^ Recently, a copper-dependent form of cell death, cuproptosis, has been recognized, which targets lipoylated TCA cycle proteins.^47^ At least two reports showed upregulation of cuproptosis-related genes in the setting of spinal cord injury,^48^ suggesting that cuproptosis may influence axon regeneration by producing cell injury and death or by metabolic consequences of upregulation of cuproptosis-related genes.

Altogether, this study demonstrates that TPEN can act as a potent intracellular copper chelator, thereby introducing uncertainties in the interpretation of TPEN-induced biological effects that have commonly been attributed to zinc. Nonetheless, TPEN remains an essential and powerful tool for studying zinc-dependent biological processes when used in conjunction with appropriate controls for specificity. By introducing the copper-selective chelator PSP-2 as a complementary tool, we demonstrate here a strategy to disentangle copper- from zinc-mediated effects, thus minimizing uncertainties in data interpretation. This approach will not only enhance the utility of TPEN in future studies but also provide a valuable tool for reassessing TPEN-induced biological effects to achieve an overall more accurate understanding of metal-regulated cellular processes and the potential involvement of copper.

## Methods

### Electrochemistry

The reduction potential of the TPEN-Cu­(II) complex was determined by cyclic voltammetry in a buffered solution at pH 5.0 (10 mM PIPBS, 0.1 M aqueous KCl) using a CH-Instruments potentiostat (model 600A). The copper complex was formed in situ by addition CuSO_4_·5H_2_O (400 μM) to an excess of TPEN (600 μM). All measurements were carried out under an atmosphere of argon in a single compartment cell with a glassy carbon working electrode, a Pt counter electrode, and an aqueous Ag/AgCl reference electrode (1 M KCl). The half-wave potentials (*E*_1/2_) were referenced to ferrocenium Fc^+/0^ couple (0.40 vs SHE)^28,49^ as external standard. The reported half-wave potentials represent the average of three independent experiments.

### Stability Constant Calculations

To calculate the Cu­(I) stability constant of TPEN, we employed the Nernst eq 1 with *E*° = 0.153 V,^28^ adjusted to 0.13 V vs SHE to account for 0.1 M ionic strength,^22,29^ and used the corresponding apparent stability constant of the Cu­(II)-TPEN complex at pH 5.0 (log*K* = 17.90) as defined by Schwarzenbach^34^ based on eq 2. The relevant protonation constants are compiled in Table 1.

2 $$K_{Cu\left(II\right)}^{\prime }=\frac{K_{Cu\left(II\right)}}{\alpha_{L}}$$

with

$$\alpha_{L}=\left[TPEN\right]+\left[{TPENH}^{+}\right]+\left[{TPENH}_{2}^{2+}\right]+\left[{TPENH}_{3}^{3+}\right]+\left[{TPENH}_{4}^{4+}\right]$$

To determine the apparent stability constant of the Cu­(I)-TPEN complex at a given pZn activity with pZn = −log­([Zn­(II)]), the original definition by Schwarzenbach was expanded according to eq 3

3 $$\alpha_{L}=\left[TPEN\right]+\left[{TPENH}^{+}\right]+\left[{TPENH}_{2}^{2+}\right]+\left[{TPENH}_{3}^{3+}\right]+\left[{TPENH}_{4}^{4+}\right]+\left[Zn\left(II\right)TPEN\right]$$

and

$$K_{Cu\left(I\right)}^{\prime }=K_{Cu\left(I\right)}/(1+10^{\left(p{Ka}_{1}-pH\right)}{+10}^{\left(p{Ka}_{1}+p{Ka}_{2}-2pH\right)}{{+10}^{\left(p{Ka}_{1}+p{Ka}_{2}+p{Ka}_{3}-3pH\right)}+10^{\left(p{Ka}_{1}+p{Ka}_{2}+p{Ka}_{3}+p{Ka}_{4}-4pH\right)})\;+10}^{\left(\log K_{Zn\left(II\right)}-pZn\right)}$$

### Spectrophotometric Competition Studies

All stock solutions and buffers were prepared with 18.2 MΩ·cm Milli-Q water and passed through a 0.2 μm membrane filter. UV–vis spectra were acquired in a quartz cuvette with 1 cm path length at 25 °C using with a CaryBio50 spectrophotometer (Agilent) equipped with temperature-controlled cuvette holder. Each experiment was repeated to verify the reproducibility.

### Competitive Removal of Cu­(I) from BCS or PSP-2 by TPEN

A solution of 100 μM BCS and 30 μM [Cu­(I)­MCL-2]­PF_6_ ^29^ was prepared in pH 7.0 buffer (10 mM PIPES, 0.1 M KCl, 25 °C). After recording an absorption spectrum from 400 to 600 nm, TPEN was added with rapid stirring from a stock solution (3 mM in 5 mM HCl) to a final concentration of 50 μM. The metal exchange reaction was followed by acquiring UV–vis traces with 6 s intervals at 5 nm spectral resolution. An identical experiment was performed in a septum-sealed cuvette under anaerobic conditions using degassed buffer solutions purged with argon for 10 min.

### Competitive Removal of Cu­(I) from PSP-2 by TPEN

A 50 μM solution of [(PSP-2)­Cu­(I)]­Cl^14^ in pH 7.0 buffer (10 mM PIPES, 0.1 M KCl, 25 °C) was supplemented with 50 μM of TPEN from a 3 mM stock solution in DMSO. The reaction was followed in a quartz cuvette with stirring. Anaerobic experiments were performed in a septum-sealed quartz cuvette analogous to the BCS competition experiment. To avoid potential interference of DMSO, TPEN was added from a 3 mM aqueous stock solution containing 5 mM HCl.

### Quantification of Cellular Metal Levels

NIH 3T3 mouse fibroblasts were grown in 15 cm Petri plastic dishes using DMEM supplemented with glucose (4.5 g/L), glutamine (584 mg/L), sodium pyruvate (110 mg/L), 10% bovine calf serum, 1% penicillin–streptomycin, and 50 μM CuSO_4_·5H_2_O. After reaching 80% confluency, the growth medium was replaced with full medium containing 10 μM of PSP-2 or TPEN (supplied from a 10 mM stock solution in DMSO) but lacking the additional CuSO_4_·5H_2_O. After incubating for 5 h, the medium was removed, and the cells were detached with trypsin and separated by centrifugation (4500 *g*, 5 min). As a control, cells were treated with an equal amount of DMSO without chelator. To assess the effect of CuGTSM, cells were preincubated with 10 μM CuGTSM for 15 min prior to the addition of 10 μM TPEN. All cell pellets were digested with 100 μL of nitric acid (67%) at 90 °C. After 2 h, 50 μL of hydrogen peroxide (30%) was added and the solution was heated for an additional hour at 90 °C. The resulting samples were diluted with 1.9 mL of ultrapure water for elemental quantification by ICP-MS (Agilent 7900). All measurements and statistical analysis are based on five independent biological repeats.

### Ratiometric Imaging of Labile Cu­(I)

Cells were grown in 35 mm glass bottom dishes (MatTek, Ashland, MA) for 2 days until confluent. The growth medium was aspirated off, and the cells were washed twice with PBS. The cells were incubated in DMEM media supplemented with 1 μM crisp-17 (supplied from a 1 mM stock solution in DMSO), 10% FBS, glucose, glutamine, HEPES, and sodium pyruvate for 20 min before imaging began. Reagents were added in 10 min intervals at the specified concentrations. The glass bottom dish was directly mounted on the microscope stage of a Zeiss LSM confocal NLO 710 microscope equipped with a femtosecond-pulsed Ti/sapphire laser. Fluorescence micrographs were acquired with excitation at 880 nm and the emission was collected from 479−536 and 611–750 nm with two bandpass filters. Ratiometric quantification of the 16 bit gray scale raw images was performed with ImageJ^50^ as described previously.^51^ For each time point, the average emission ratio was analyzed for 10 cells.

### Animal Use, Surgeries, and Intraocular Injections

Animal studies were performed at Boston Children’s Hospital with approval of the Institutional Animal Care and Use Committee. Mice (129S1/SvlmJ; Jackson Laboratory; #002448) were housed in the animal facility with a 12 h light/12 h dark cycle (lights on from 7:00 AM to 7:00 PM) and a maximum of five adult mice per cage. Animals were assigned to different treatment groups and, in any given experiment, surgeries were done for several groups at a time. Optic nerve crush (ONC) surgeries were carried out on male mice 8 week of age (average body weight, 20–26 g) under general anesthesia, as described previously.^19^ TPEN (100 μM; the dose was informed by previous studies),^9^ or PSP-2 (50 μM) or PSP-2 preloaded with Cu­(I) (50 μM) or vehicle (1% DMSO in 0.9% saline) were injected intraocularly immediately after ONC. Subsequent processing was performed blinded to treatment.

### Quantitation of Retinal Ganglion Cell Survival and Axon Regeneration

Retinal ganglion cell (RGC) survival and axon regeneration were quantified as in earlier studies from our laboratory and others.^17,52^ Mice were given an overdose of anesthesia 2 weeks after ONC and perfused transcardially with isotonic phosphate buffered saline and 4% (wt/vol) paraformaldehyde (PFA). Whole eyes and optic nerves were dissected and postfixed in 4% PFA for 2 h. Retinas were dissected and immunostained as whole-mounts with an antibody to βIII-tubulin (1:500, rabbit polyclonal; Abcam, #ab18207), followed by an Alexa Fluor 488-conjugated secondary antibody to rabbit IgG, taking advantage of the selective expression of βIII-tubulin in RGCs in the ganglion cell layer. ImageJ software was used to count βIII-tubulin positive cells from eight fluorescently illuminated images per retina (Nikon 80i) taken at prespecified areas, four at 1 mm and four at 2 mm from the optic nerve head, then averaged to estimate overall RGC survival per square millimeter.

Optic nerves were cryopreserved by incubation in 30% sucrose, embedded in OCT Tissue Tek Medium (Sakura Finetek), frozen, cryostat-sectioned longitudinally at 10 μm, and mounted on glass slides. Regenerating axons were labeled by immunostaining with a GAP-43 antibody (in-house made in sheep; 1:2000 ^53^) followed by Alexa Fluor 488-conjugated secondary antibody to sheep IgG. The optic nerves were imaged and the axons were counted manually under the microscope (Nikon 80i) in eight longitudinal sections per case at prespecified distances from the ONC site (0.5 mm, 1 mm), and the total number of regenerating axons per nerve was estimated.

Statistical analysis was performed using the GraphPad Prism 10 for MacOS statistical package. Tests of normality were applied, and not all data sets appeared drawn from a normally distributed population. Therefore, the nonparametric one way ANOVA Kruskal–Wallis test followed by Dunn’s Multiple Comparisons test was used to assay significance of differences between conditions.

## §

Department of Ophthalmology, University of Pittsburgh, Pittsburgh, Pennsylvania 15219, United States

## Abbreviations

TPEN: *N,N,N′,N′*-tetrakis­(2-pyridylmethyl)­ethylenediamine
CNS: central nervous system
ONC: optic nerve crush
GAP-43: antigrowth associated protein-43
PIPBS: piperazine-*N,N′-*bis­(4-butanesulfonic acid)
SHE: standard hydrogen electrode
BCS: bathocuproine disulfonate
EGTA: ethylene glycol-bis­(2-aminoethyl ether)-*N,N,N′,N′*-tetraacetic acid
CuGTSM: copper­(II), glyoxal-bisN­(4)-methylthiosemicarbazone
DMEM: Dulbecco′s Modified Eagle′s Medium
HEPES: 4-(2-Hydroxyethyl)­piperazine-1-ethanesulfonic acid
DMSO: dimethyl sulfoxide
PFA: paraformaldehyde
OCT: optimal cutting temperature (compound)
FBS: fetal bovine serum
